# Super recognizers: Increased sensitivity or reduced biases? Insights from serial dependence

**DOI:** 10.1167/jov.24.7.13

**Published:** 2024-07-24

**Authors:** Fiammetta Marini, Mauro Manassi, Meike Ramon

**Affiliations:** 1 School of Psychology, University of Aberdeen, King's College, Aberdeen, UK; 2 Applied Face Cognition Lab, Institute of Psychology, University of Lausanne, Lausanne, Switzerland; 3 AIR – Association for Independent Research, Zürich, Switzerland

**Keywords:** serial effects, sequential effects, super recognizers, priming, visual processing, sensitivity versus bias

## Abstract

Super recognizers (SRs) are people that exhibit a naturally occurring superiority for processing facial identity. Despite the increase of SR research, the mechanisms underlying their exceptional abilities remain unclear. Here, we investigated whether the enhanced facial identity processing of SRs could be attributed to the lack of sequential effects, such as serial dependence. In serial dependence, perception of stimulus features is assimilated toward stimuli presented in previous trials. This constant error in visual perception has been proposed as a mechanism that promotes perceptual stability in everyday life. We hypothesized that an absence of this constant source of error in SRs could account for their superior processing—potentially in a domain-general fashion. We tested SRs (*n* = 17) identified via a recently proposed diagnostic framework (Ramon, 2021) and age-matched controls (*n* = 20) with two experiments probing serial dependence in the face and shape domains. In each experiment, observers were presented with randomly morphed face identities or shapes and were asked to adjust a face's identity or a shape to match the stimulus they saw. We found serial dependence in controls and SRs alike, with no difference in its magnitude across groups. Interestingly, we found that serial dependence impacted the performance of SRs more than that of controls. Taken together, our results show that enhanced face identity processing skills in SRs cannot be attributed to the lack of serial dependence. Rather, serial dependence, a beneficial nested error in our visual system, may in fact further stabilize the perception of SRs and thus enhance their visual processing proficiency.

## Introduction

Super recognizers (SRs) are individuals who exhibit naturally occurring superiority for processing facial identity. Russell, Duchaine, and Nakayama (2009) initially coined the term *SR* when they documented the exceptional abilities of four individuals in various tasks such as matching unfamiliar faces, remembering unfamiliar faces, and identifying celebrities based on their childhood photographs. Despite limited knowledge concerning the abilities of SRs, law enforcement agencies internationally are increasingly interested in their deployment to improve their operations (Dunn, Towler, Kemp, & White, 2023; Hucklenbroich, 2023; Mayer & Ramon, 2023; Moreton, Pike, & Havard, 2019; Ramon, Bobak, & White, 2019a; Ramon, Bobak, & White, 2019b; Ramon, 2021; rbb Ferneshen Berlin, 2023). In the last decade, research into SRs has increased, particularly investigating how they should be identified (Ramon, 2021), and the nature or underlying mechanisms of their unique ability (Linka, Broda, Alsheimer, de Haas, & Ramon, 2022; Nador, Vomland, Thielgen, & Ramon, 2022; Nador, Zoia, Pachai, & Ramon, 2021).

At its core, the enhanced face processing abilities of SRs could be due to two main factors. On the one hand, their performance could be due to enhanced perceptual sensitivity to face stimuli. Recent work reporting increased visual salience for face stimuli in SRs supports this view (Linka et al., 2022). Another possibility is that the performance of SRs may be less impacted by perceptual biases known to affect performance in neurotypical observers. For example, visual perception is characterized by several kinds of misperceptions related to the stimuli (Kosovicheva & Whitney, 2017; Wang et al., 2022; Wang, Murai, & Whitney, 2020), spatial context (Awad, Clifford, White, & Mareschal, 2020; Bar & Ullman, 1996; Manassi & Whitney, 2018), and temporal context (Fischer & Whitney, 2014; Kohn, 2007; Liberman, Fischer, & Whitney, 2014; Webster, 2011) to which we are exposed on a regular basis (Schwartz, Hsu, & Dayan, 2007). It is thus conceivable that the enhanced performance of SRs could be attributed to a lack of such biases, which in turn could support the rapid formation of robust, invariant facial representations across highly varied input conditions (Dunn et al., 2022; Nador et al., 2021; Nador et al., 2022).

In our study, we investigated whether the enhanced face-identity processing skills of SRs coincide with the lack of visual biases occurring in the time domain, specifically positive serial dependence. Positive serial dependence refers to a phenomenon in which actions, perceptions, decisions, and memories of features or objects are systematically biased toward the recent past (Fischer & Whitney, 2014; Manassi, Murai, & Whitney, 2023; Pascucci et al., 2023; Whitney, Manassi, & Murai, 2022). This attractive bias is in contraposition with negative aftereffect (Fritsche, Mostert, & de Lange, 2017; Kohn, 2007; Rafiei, Hansmann-Roth, Whitney, Kristjánsson, & Chetverikov, 2021; Webster, 2015), a known form of repulsive/negative bias. Serial dependence occurs across a large variety of stimuli (Manassi et al., 2023) and can manifest as a misperception of current stimuli toward the past (Cicchini, Mikellidou, & Burr, 2017; Cicchini, Mikellidou, & Burr, 2018; Manassi & Whitney, 2022). Given its significance and ubiquity, serial dependence has been extensively studied over the past decade (Manassi et al., 2023; Pascucci et al., 2023) and has been proposed as a mechanism that promotes perceptual stability in everyday life (Manassi & Whitney, 2022; Manassi & Whitney, 2024).

Here, we hypothesized that diminished serial dependence for facial identity could be related to, and therefore account for, SRs’ superiority for processing facial identity. To anticipate our results, we found that serial dependence occurred in the facial identity and shape perception domains and exhibited comparable magnitude across groups. Intriguingly, we found a stronger relationship between magnitude of serial dependence and individuals’ performance among SRs as compared with control observers.

## General methods

This research was approved by the Ethics Committee of the School of Psychology of the University of Aberdeen (UK) and of the University of Fribourg (CH). Informed consent was obtained from participants at the beginning of the study. All experimental procedures followed the tenets of the Declaration of Helsinki.

### Participants

#### Experimental group

We recruited 17 SRs identified via a recently proposed formal diagnostic framework for SR identification (Ramon, 2021). Their face perception abilities were tested with the 40-item long form of the Yearbook Test (YBT) (Bruck, Cavanagh, & Ceci, 1991; Fysh, Stacchi, & Ramon, 2020; Stacchi, Liu-Shuang, Ramon, & Caldara, 2019) and the Facial Image Card Sorting Test (FICST) (Fysh et al., 2020; Jenkins, White, Van Montfort, & Burton, 2011; Stacchi et al., 2019). Their face recognition abilities were tested with the long version of the Cambridge Face Memory Test (CFMT+) (Russell et al., 2009). Individuals were considered as SRs if they scored above a cut-off in at least two of these tests (Ramon, 2021). We excluded from analyses participants with (1) response errors that exceeded 3 *SD* from the individual mean in more than 1/4 of the trials (thus suggesting random responses); or (2) average adjustment times longer than 15 seconds (reaction time cut-off) in one of the two sessions of the study. Two participants were excluded due to the second exclusion criterion. In total, the data of 15 SRs (seven females; mean age = 35.4 years, *SD* = 5.6 years) were analyzed. All SRs received a €50 Amazon gift voucher as an appreciation of their participation.

#### Control group

Twenty participants were recruited for the experiment by using the Prolific platform (https://www.prolific.co/). Two participants were excluded from the analyses because they dropped out in the second session of the experiment; another was excluded due to the first exclusion criterion. In total, the data of 17 control participants (eight females; mean age = 33.6 years, *SD* = 5.5 years) were analyzed. Participants were German native speakers and received a total of €30 (15€/hr) for their participation.

### General procedure

The study consisted of two experimental sessions of approximately 60 minutes each. The task was to adjust a facial identity to match a previously seen one (face identity adjustment task, Experiment 1), and adjust a shape to match the appearance of the one previously displayed (shape adjustment task, Experiment 2). All of the experiments were designed in the PsychoPy experiment Builder (Peirce et al., 2019). Participants completed the experiments online with their personal computers through the Pavlovia platform (http://www.pavlovia.org/) and were asked to use the Google Chrome browser. At the beginning of each session, participants were required to calibrate their screen by adjusting an image of a credit card to the size of a real credit card (85.6 mm × 53.98 mm). This procedure allowed us to ensure that the stimuli were shown in proportion to the personal computer monitor of each participant. Before starting the experiment, each participant completed a block of 15 practice trials, during which feedback was provided. The order of the two experiments was counterbalanced across participants. Participants’ data are available on the OSF website at the link: https://osf.io/xkhu6/?view_only=70c4167090014d4bbb9fbced4a83e507.

## Experiment 1: Face identity adjustment task

### Material and stimuli

**Figure 1.**
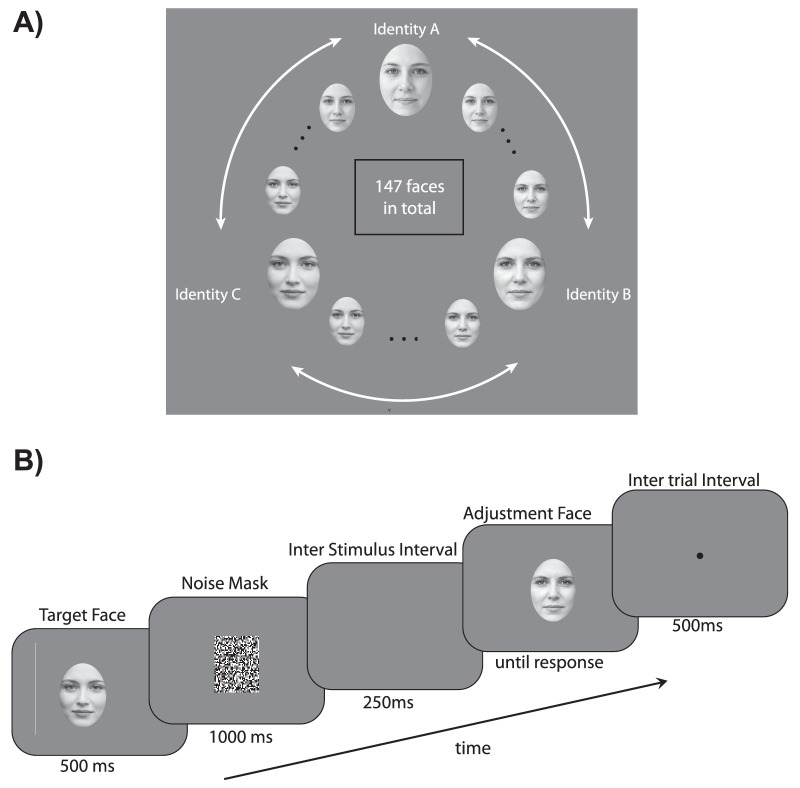
Experiment 1: Stimuli and trial sequence. (**A**) A circular face morph continuum was created among three original prototype shapes (identities **A**, **B**, and **C**), resulting in 147 face morphs. (**B**) On each trial, a random target face (500 ms) was presented at the center of the screen, followed by a noise patch, ISI, and finally the adjustment face. Participants were asked to adjust its appearance to match the identity of the previously seen face. Faces in the figure are not the stimuli displayed during the experiment, but they were artificial intelligence-generated for illustrative purposes only.

Observers were presented with a series of neutral grayscale face images cropped in an oval shape and displayed at the center of the screen. Face stimuli size was 0.4 (width) by 0.5 (height) PsychoPy “height” units; this kind of unit refers to the stimulus size in relation to the aspect ratio of the window. The faces were randomly extracted from a face morph continuum created among three Caucasian female Ekman identities (A, B, and C), with permission (Ekman & Friesen, 1976). A set of 48 face morphs was generated between each pair of identities, resulting in a morph wheel of 147 face images (Figure 1A). After each face, a black and white pixels noise mask of 0.4 (width) by 0.5 (height) PsychoPy “height” units was displayed in the middle of the screen right after face target presentation. Participants used a keyboard for responding (left/right arrow keys; see adjustment task below). Stimuli were displayed on a gray background.

### Procedure

On each trial, participants viewed a 500-ms face randomly selected from the identity morph wheel in the center of the screen (Figure 1B). Next, a patch of noise appeared for 1000 ms, and after a 250-ms interstimulus interval (ISI) another randomly selected face was presented at the center of the screen. The participants’ task was to adjust the appearance of the test face with the left and right keyboard arrows to match the identity of the previously seen target face (face identity adjustment task). After the face adjustment, participants pressed the space bar to confirm their response, and following a 500-ms intertrial interval, the next trial started. Participants completed eight blocks of 50 trials each, resulting in a total of 400 trials. At the end of each block, participants were told to take a break and proceed to the next block by pressing the space bar.

### Analyses

#### Serial dependence analysis

The perceptual error in the face adjustment task was calculated as the shortest distance along the morph wheel between the match and target faces (adjustment response, with the face morph displayed in the current trial). For each observer, we excluded from the analysis the trials in which the absolute response error exceeded 3 *SD* from the mean error in the adjustment task across the whole experiment. In addition, we removed trials with a response time longer than 15 seconds. Following these two criteria, no more than 5% of the trials were removed per observer. Each participant's error on a given trial was compared to the difference in target face identities between the current and previous trial (face morph displayed in the previous trial vs. face morph displayed in the current trial). We then fit a simplified derivative of Gaussian (DoG) to the running average of each participant's data in the form:

$$y=abcxe^{-\left(bx\right)2}$$

**Figure 2.**
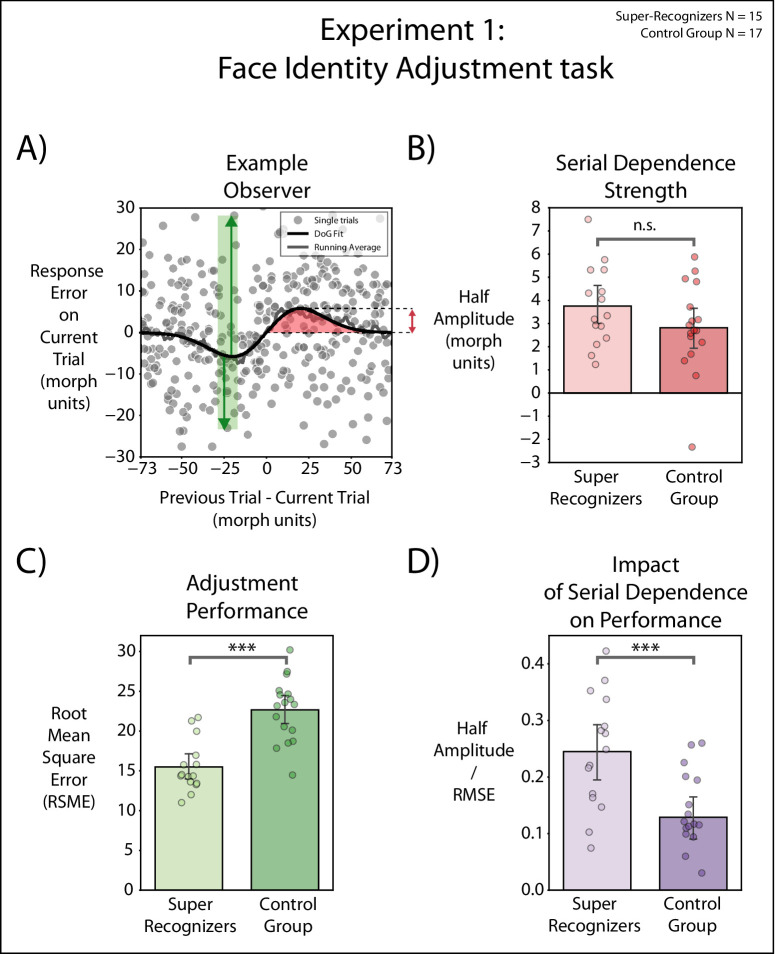
Experiment 1. (**A**) Example observers. In units of face morph steps, the *x*-axis is the shortest distance along the morph wheel between the current and one-back faces, and the *y*-axis (response error) is the shortest distance along the morph wheel between the selected match face and current target face. Positive *x*-axis values indicate that the one-back face was clockwise on the face morph wheel relative to the current face, and positive *y*-axis values indicate that the current adjusted face was also clockwise relative to the current face. Each data point shows performance on one trial. To quantify the magnitude of serial dependence, we fit a DoG to the data (black line) measuring the half-amplitude peak for each observer. The red arrow indicates the half-amplitude peak of the DoG fit. The green arrows indicate the adjustment error from the DoG fit (RMSE). (**B**) Serial dependence strength for one-back. Half amplitudes of DoGs for one-trial back. Each filled dot represents the half amplitude (morph units) for a single observer. Bars indicate the mean for the two groups (SRs and controls), and error bars are standard errors of the mean. (**C**) Adjustment performance. Each filled dot shows the RMSE from the DoG fit for a single participant. Bars indicate the means for the two groups. Error bars indicate the standard errors of the mean for each group. (**D**) Serial dependence as a function of adjustment performance. We calculated the ratio between serial dependence strength (half amplitude of DoG fit) and adjustment performance (RMSE). Bars indicate the means for each group. Error bars indicate the standard errors of the mean for each group.

In this formula, *y* is the relative response error for each trial, which refers to the relative shortest distance along the morph wheel between the target stimulus and the response stimulus; *x* represents the shortest distance along the morph wheel between the target stimulus presented in the previous trial and the test stimulus presented in the current trial; *a* represents the half amplitude of the DoG curve; *b* is an index of the width of the curve derivative; and, finally, *c* is a constant scaling factor √2/e^–^^0.5^ that makes the *a* parameter equal to the half-amplitude curve peak. A constrained nonlinear minimization of the residual sum of squares was used to fit the DoG on participants’ data. As an index of the strength and direction of the attraction, we considered the half amplitude (*a*) of the DoG curve (Figures 2A and 2B). A positive half-amplitude value indicates that a perceived face identity on a given trial was attracted in the direction of the previously seen target face appearance, whereas a negative half-amplitude value indicates that a perceived face identity was pulled away from the previously seen target stimulus appearance. A half amplitude of zero is indicative of no bias in the perception of the present stimulus.

#### Adjustment performance

To obtain a measure of adjustment performance independent on serial dependence biases, we computed the root mean square error (RMSE) on the DoG curve we previously fitted for each observer (Figure 2C). This measure (Cicchini et al., 2018; Cicchini, D'Errico, & Burr, 2022), unlike the response error, yields a measure of adjustment performance independently of temporal biases (i.e., serial dependence or negative aftereffect). To further quantify discriminability of the stimuli, we fit a von Mises function to each observer's response error frequency distribution and computed the corresponding cumulative distribution function (CDF). For each observer's individual CDF, a continuous report discrimination (CRD) index was defined as half of the difference between the 25th and 75th percentile of their CDF. This measure can be considered as the equivalent of just noticeable difference for continuous reports (for a detailed explanation of the analysis, see figure 2 in Manassi et al, 2021).

#### Impact of serial dependence on adjustment performance

To quantify the impact of serial dependence on participants’ performance (Figure 2D), we calculated the ratio between half amplitude of the DoG fit (strength of serial dependence) and the RMSE (its corresponding adjustment performance).

### Results

First, we investigated serial dependence strength across groups by calculating the half amplitude of the DoG curve fitted to participants’ data (see example observer in Figure 2A). All SRs and control participants (bar one) displayed a positive DoG half amplitude (Figure 2B, red bars), suggesting that, in both groups, observers’ perception of the facial identity on a given trial was attracted toward the facial identity presented in the previous trial. Serial dependence was present in both SRs, *t*(14) = 8.47 (*p* < 0.001, one-sample *t*-test), and the control group, *t*(16) = 6.02 (*p* < 0.001, one-sample *t*-test). As shown in Figure 2B, there was no significant difference in the serial dependence strength between the SRs (half-amplitude mean = 3.75, *SE* = 0.44) and the control group (half-amplitude mean = 2.81, *SE* = 0.46), *t*(30) = 1.44 (*p* = 0.16, two-sample *t*-test).

Second, as a measure of observers’ adjustment performance, we computed the RMSE of the DoG fit (Figure 2C, green bars). This measure yielded an index of the adjustment performance in the task, independently of temporal biases such as serial dependence or negative aftereffect. As expected from previous literature on SRs, we found a significant difference in adjustment performance between the SRs (RMSE = 15.48, *SE* = 0.82) and the control group (RMSE= 22.65, *SE* = 0.96), *t*(30) = –5.56 (*p* < 0.001, two-sample *t*-test), thus showing face superiority among the SR group. The *R*^2^ from the DoG fit in both groups yielded similar results (SR mean = 0.9735, *SE* = 0.005; control mean = 0.9893, *SE* = 0.002), *t*(30) = –2.82 (*p* = 0.008, two-sample *t*-test). As a further measure of discriminability, the mean CRD index was also significantly lower in SRs compared with controls (6.47 ± 0.87 morph units for SRs and 8.92 ± 1.81 morph units for controls), *t*(30) = –4.75 (*p* < 0.001).

Third, we investigated the impact of serial dependence on performance across the two groups (Figure 2D, violet bars). In fact, we considered the strength of serial dependence as a percentage of the specific performance of each observer. To this purpose, we computed the ratio between serial dependence strength (as exhibited by half amplitudes of serial dependence strength) and adjustment performance (as shown by RMSE on the DoG fit). High values indicate that serial dependence impacted performance more, whereas low values indicate that serial dependence impacted performance less. We found a significant difference between SRs and controls, as serial dependence had a greater impact on the performance of SRs compared with controls, *t*(30) = 3.49 (*p* = 0.001).

Taken together, the results of Experiment 1 show that (1) SRs and controls exhibited serial dependence in face perception (even in an online experimental setting), and that (2) serial dependence did not differ across groups, suggesting that SRs’ enhanced facial identity processing cannot be attributed to a lack of temporal sequential biases (i.e., serial dependence). Interestingly, when calculating serial dependence strength as a ratio of performance, we found that (3) the performance of SRs was more greatly impacted by serial dependence compared with that of controls.

## Experiment 2: Shape adjustment task

Experiment 1 showed that serial dependence impacts adjustment performance more in SRs than control observers. As SRs in our sample were identified based on their enhanced face identity processing abilities, we predicted that this increased impact of serial dependence would not be observed for other stimuli (e.g., shapes). To this end, we investigated the strength of serial dependence for shape perception in SRs and controls.

### Material and stimuli

**Figure 3.**
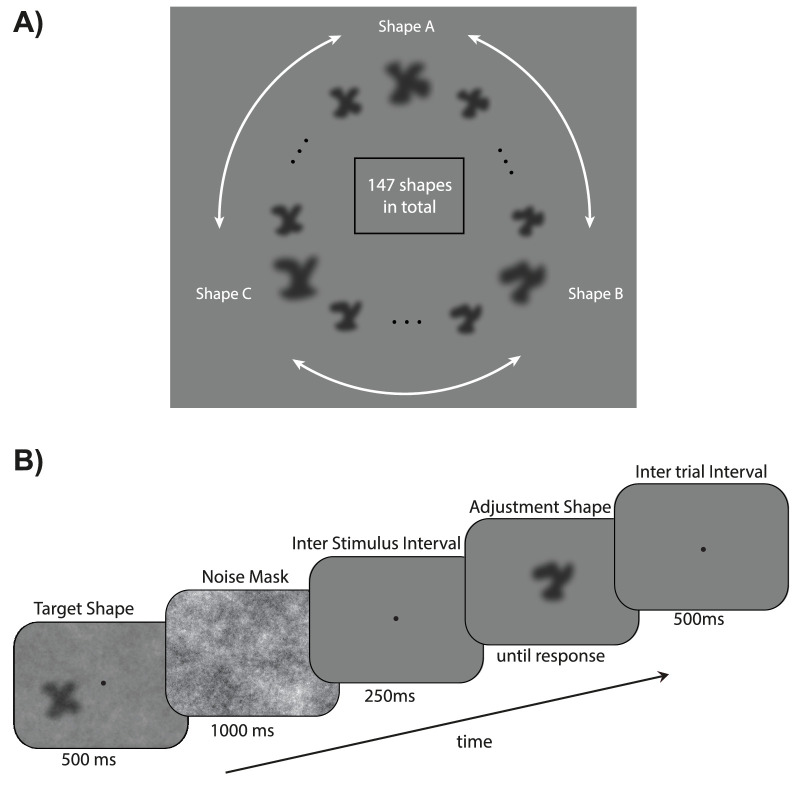
Experiment 2: Stimuli and trial sequence. (**A**) A circular shape morph continuum was created among three original prototype shapes, resulting in 147 shape morphs. (**B**) On each trial, a random target shape was presented in the center of the screen, followed by a noise mask. After an ISI, a test shape was shown, and participants were asked to adjust its appearance to match the shape of the previously seen stimulus. The next trial started after an intertrial interval following confirmation of a response.

Observers were the same as in Experiment 1. Apparatus and procedure were the same as Experiment 1 but with the following differences. Instead of face identities, the stimuli were grayscale shapes of [0.5, 0.5] PsychoPy “height” units. A set of 48 shape morphs was generated among three original prototype shapes (shapes A, B, and C), resulting in a morph wheel of 147 images (Manassi, Kristjánsson, & Whitney, 2019; Manassi et al., 2021) (Figure 3A). This set of stimuli and paradigm was previously used to probe the strength of serial dependence in shape perception (Manassi et al., 2019). In addition, instead of a black-and-white pixel noise mask in the middle of the screen, in this experiment we presented a noise mask that covered the entire screen. Similarly to Experiment 1, on each trial participants were asked to adjust the appearance of a test shape to match the target shape of the previously seen object (Figure 3B).

### Results

**Figure 4.**
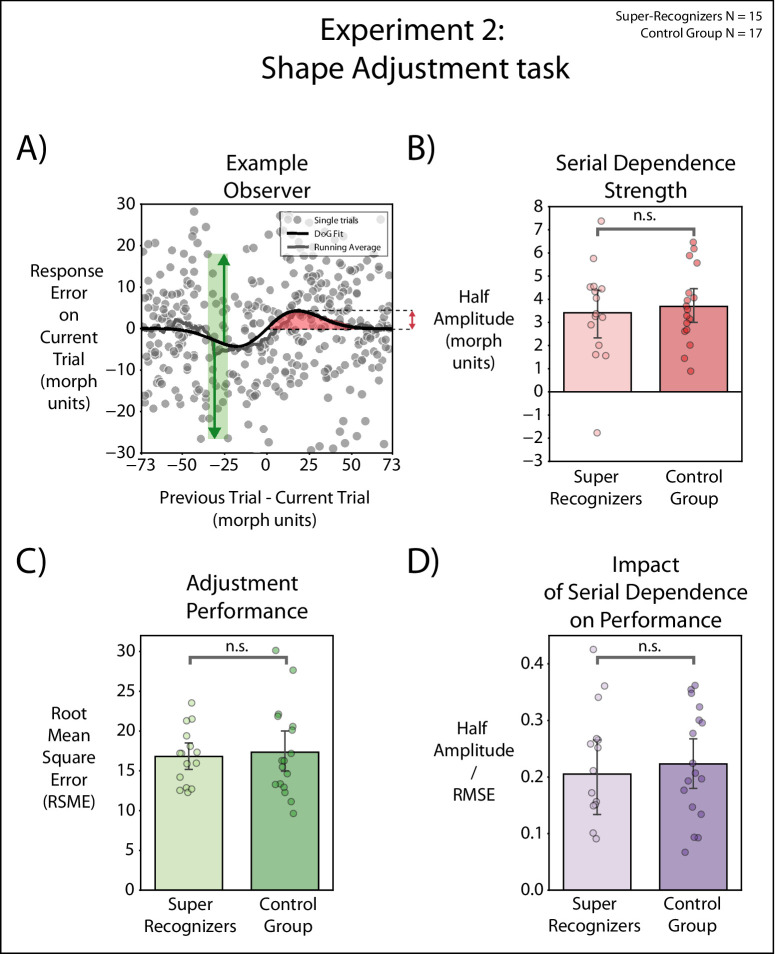
(**A**) Example observers. In units of shape morph steps, the *x*-axis is the shortest distance along the morph wheel between the current and one-back shapes, and the *y*-axis (response error) is the shortest distance along the morph wheel between the selected match shape and current target shape. Positive *x*-axis values indicate that the one-back shape was clockwise on the shape morph wheel relative to the current shape, and positive *y*-axis values indicate that the current adjusted shape was also clockwise relative to the current face. Each data point shows performance on one trial. To quantify the magnitude of serial dependence, we fit a DoG to the data (black line) measuring the half-amplitude peak per observer. The red arrow indicates the half-amplitude peak of the DoG fit. The green arrows indicate the adjustment error from the DoG fit (RMSE). (**B**) Serial dependence strength for one-back. Half amplitudes of DoG for one-trial back. Each filled dot represents the half amplitude (morph units) for a single observer. Bars indicate the means for the two groups, and error bars are standard errors of the mean. (**C**) Adjustment performance. Each filled dot shows the RMSE from the DoG fit for a single participant. Bars indicate the means for the two groups. Error bars indicate the standard errors of the mean for each group. (**D**) Serial dependence as a function of adjustment performance. We calculated the ratio between serial dependence strength (half amplitude of DoG fit) and adjustment performance (RMSE). Bars indicate the means for the two groups, and error bars indicate the standard errors of the mean for each group.

First, we investigated serial dependence strength in both groups by calculating the half amplitude of the DoG curve fitted to participants’ data (Figures 4A and 4B). All SRs and all control group participants displayed a positive DoG half-amplitude (Figure 4B, red bars), suggesting that in both groups observers’ perception of the shape on a given trial was attracted toward the shape presented in the previous trial. Serial dependence was present in both SRs, *t*(14) = 6.28 (*p* < 0.001, one-sample *t*-test against zero) and controls, *t*(16) = 9.42 (*p* < 0.001, one-sample *t*-test). There was no significant difference in serial dependence magnitude between the SRs (half-amplitude mean = 3.41, *SE* = 0.54) and controls (half-amplitude mean = 3.68, *SE* = 0.39), *t*(30) = –0.41 (*p* = 0.68, two-sample *t*-test).

Second, as a measure of observers’ adjustment performance, we computed the RMSE of the DoG fit (Figure 4C). This measure yielded an index of response error performance, independently of temporal biases such as serial dependence or negative aftereffects. We found no significant difference in performance between the SRs (RMSE = 16.80, *SE* = 0.91) and the control group (RMSE = 17.34, *SE* = 1.38), *t*(30) = –0.32 (*p* = 0.75, two-sample *t*-test), thus indicating no shape perception superiority for SRs. The *R*^2^ from the DoG fit in both groups yielded similar results (SR mean = 0.973, *SE* = 0.005; control mean = 0.9723, *SE* = 0.004), *t*(30) = 0.10 (*p* = 0.92, two-sample *t*-test). Furthermore, the mean continuous report discrimination index was not significantly different in SRs compared with controls (7.06 ± 1.16 morph units for SRs and 7.10 ± 1.99 morph units for controls), *t*(30) = –0.07 (*p* = 0.93). Overall, these results revealed no difference in accuracy for SRs’ and controls’ shape adjustment performance.

Third, we calculated the impact of serial dependence on shape adjustment performance across both groups (Figure 4D). To this end, we computed the ratio between serial dependence strength (as exhibited by half amplitudes of serial dependence strength) and adjustment performance (as shown by RMSE on the DoG fit). We found no significant difference between SRs and controls, as serial dependence had a similar and comparable impact on the performance of both SRs and controls, *t*(30) = –0.43 (*p* = 0.66).

Taken together, the results from Experiment 2 show that (1) SRs and controls exhibited serial dependence in shape perception, and (2) this attractive bias did not differ across groups. When calculating serial dependence strength as a ratio of their shape adjustment performance, we found that (3) the performance of SRs was as similarly impacted by serial dependence as the performance exhibited by controls.

## General discussion

Notwithstanding the growing empirical evidence regarding the visual and cognitive abilities of SRs, two aspects remain to be clarified. First, the mechanisms underlying SRs’ processing superiority are currently being researched and therefore remain unspecified. Second, it is unknown whether the superiority of SRs is confined to the category of faces (see, e.g., Bobak, Bennetts, Parris, Jansari, & Bate, 2016; Faghel-Soubeyrand et al., 2024; Nador & Ramon, 2022). Here, for the first time to our knowledge, we sought to address both questions psychophysically, by investigating serial dependence mechanisms for face identity and shape processing in both non-SRs and SRs. Our working hypothesis was that the processing superiority of SRs may be attributed to the lack of temporal biases, serial dependence in particular (Fründ, Wichmann, & Macke, 2014; Manassi et al., 2023).

Across both experiments, SRs and controls exhibited serial dependence of comparable magnitude for both face identity (Experiment 1) and shape processing (Experiment 2). However, subsequent analyses investigating the relationship between serial dependence and performance more closely revealed a difference between SRs and controls. Specifically, when computing serial dependence as a function of stimulus discriminability, we found that serial dependence had a larger impact on the performance of SRs as compared to that of the controls in the face identity adjustment task but not in the shape adjustment task. This means that SRs are more greatly impacted by serial dependence *relative to their discriminability ability*.

Recent literature has extensively investigated the relationship between serial dependence and stimulus discriminability—more specifically, noise and uncertainty. The majority of empirical evidence suggests that serial dependence increases as discriminability decreases (Gallagher & Benton, 2022; Manassi & Whitney, 2022; Manassi, Liberman, Kosovicheva, Zhang, & Whitney, 2018a; Pascucci et al., 2019; van Bergen & Jehee, 2019; but see Kim & Alais, 2021; Tanrikulu, Pascucci, & Kristjánsson, 2023). Accordingly, this property of serial dependence has been interpreted in the context of Bayesian modeling, considering past stimuli as priors for present perception (Fritsche, Spaak, & de Lange, 2020; van Bergen & Jehee, 2019). Our results show a different trend: Whereas SRs outperformed controls in terms of face discriminability, their serial dependence magnitude was equal or even lower than that exhibited by controls (Figures 2B and 2C). If this represented a characteristic feature of the SR population, it would indicate differential impact of priors related to previous stimuli. It might be argued that serial dependence is not stimulus specific; that is, this attractive bias occurs with the same strength independently of the visual stimuli (Ceylan, Herzog, & Pascucci, 2021). However, stimulus discriminability (Gallagher & Benton, 2022; Kim & Alais, 2021; Manassi & Whitney, 2022; Manassi et al., 2018a; Pascucci et al., 2019; van Bergen & Jehee, 2019) does seem to play a role in modulating serial dependence in the normal population. The fact that stimulus discriminability does not impact the processing by SRs of face stimuli may indicate that it is governed by different underlying mechanisms.

Whereas Experiment 1 showed a face superiority effect in SRs in their adjustment performance, Experiment 2 yielded no significant difference between groups. At first sight, one could interpret this as supporting the notion that the increased perceptual abilities of SRs are face specific. However, it is possible that SRs would exhibit domain-general performance superiority in the context of other experimental paradigms. For example, Faghel-Soubeyrand et al. (2024) reported that single-trial electroencephalogram responses elicited by face and non-face objects could be used to classify observers as SRs or controls.

Adding to previous findings (Bosch, Fritsche, Utzerath, Buitelaar, & de Lange, 2022; Lieder et al., 2019; Manassi et al., 2021; Ren, Li, Pietralla, Manassi, & Whitney, 2023; Stein et al., 2020), the present results reinforce the idea of serial dependence as a stable (Kondo, Murai, & Whitney, 2022; Trübutschek & Melloni, 2023), pervasive (Manassi, Murai, & Whitney, 2018b), and general mechanism across different stimuli, tasks, and populations (Manassi et al., 2023). Importantly, strictly speaking, this does not imply that serial dependence is expressed in a uniform, *fixed* manner across different types of stimuli. Indeed, it manifests differently across modalities (action, perception, decision, and memory) and levels of visual processing, from low-level ones (Goettker & Stewart, 2022; Manassi & Whitney, 2022; Murai & Whitney, 2021) to high-level ones (Barbosa et al., 2020; Ceylan, Herzog, & Pascucci, 2021; Pascucci et al., 2023). Functionally, serial dependence has been proposed as a mechanism promoting perceptual stability across a constantly changing environment providing noisy visual input (Cicchini et al., 2018; Fischer & Whitney, 2014; Manassi & Whitney, 2022; Manassi & Whitney, 2024). However, serial dependence is not always a beneficial and efficient strategy (Cicchini et al., 2018; Manassi & Whitney, 2022); in non–auto-correlated situations, such as radiological screening, it can be very detrimental to discrimination performance within clinical task settings (Manassi et al., 2019; Manassi et al., 2021; Manassi & Whitney, 2024).

The stronger relationship for SRs between discriminability and serial dependence could reinforce the idea of serial dependence as a beneficial nested error, effectively reducing the noise in the environment. As a result, SRs may exhibit enhanced performance by virtue of greater serial dependence relative to their discriminability, not despite it. We consider our results as a promising first step for this proposal, and future studies adopting an individual differences approach are required to characterize the relationship between serial dependence and perceptual discriminability with greater detail.

However, one could argue that superior processing in SRs may not be (solely) attributable to serial dependence but may rather reflect (a combination of) other perceptual biases. Indeed, various biases continuously shape our perception, including negative adaptation (Webster, 2011; Webster, 2012; Webster, 2015), stimulus-related biases (Kosovicheva & Whitney, 2017; Wang, Murai, & Whitney, 2020; Wang et al., 2022), and contextual biases (Albright & Stoner, 2002; Awad et al., 2020; Pilz & Lou, 2022), as well as biases on an individual basis (Cappe, Clarke, Mohr, & Herzog, 2014; Grzeczkowski, Clarke, Francis, Mast, & Herzog, 2017; Shaqiri et al., 2019). Extending the present findings, it is possible that SRs may exhibit differences in these effects, which could advance our understanding of mechanisms underlying their perceptual superiority.

## Conclusions

To study the mechanisms underlying the novel phenomenon of SRs, we conducted the first ever investigation of serial dependence in this unique population. Our study adds to the extremely limited psychophysical findings reported to date for SRs (Nador et al., 2021; Dunn et al., 2022). Here, we demonstrate that SRs’ superiority for facial identity extends to an adjustment task, in a paradigm which is usually designed to measure observers’ perceptual biases (Fritsche et al., 2017; Cicchini et al., 2017). Importantly, our findings were obtained under online testing conditions, without control of parameters that are typically strictly controlled in laboratory settings. This indicates that, provided sufficient participant motivation and compliance, (certain types of) psychophysical testing need not necessarily be conducted under laboratory conditions. SRs recruiting and research has been limited due to their rarity, and thus an online setting may be key in future studies for a more extensive testing.
